# 

**Published:** 1917-02-24

**Authors:** 


### CONTENTS.

PAGE

**John Hunter and War Surgery** ... ... ... 413

**The Employment of Consumptives** ... ... 414

**Hospital and Institutional News** ... ... ... 415

Sir Cooper Perry's Accident—Ophthalmia Neonatorum—The Work of a Great Manchester Hospital—A Late Director-General of the Indian Medical Service—Death of a Well-known Ophthalmic Surgeon—The Cat as a Carrier of Disease—When Doctors Differ—Coal Difficulties at Guy's—Recognition for Philanthropic Workers—The Bishop of St. Asaph on War Memorials—An Objectionable Expedient—An Expedient to Increase the Number of Beds—Misunderstandings at Merthyr—The Medical Examination of R.A.M.C. Officers—Hospitals and the Use of Medicinal Glycerine—An Heroic Kitchen-Boy—Private Charity and Public Registration—Who Should be First?—Sausages Under Surveillance—Welfare Work in Monmouthshire—Food Regulations for Institutional Staffs—What Shall We Eat?—Municipalities May Pay the Doctor—More Supervision of Midwives—Claims for Rebate of Spirit Duty—This Week's Drug Market.

**The Prevention of Venereal Disease** ... ... 421

The Ethics of the New Criminal Law Amendment Bill.

**The Sanitation of Camps** ... ... ... ... 422

By Surgeon-General Sir Alfred Keogh, G.C.B.

PAGE

**National Insurance Act Developments...** ... 423

The Final Report of the Committee of Inquiry.

**Hospital Architecture and Construction** ... 425

Addenbrooke's Hospital, Cambridge.

**War Savings Associations** ... ... ... ... 427

The Action of Two Great Hospitals.

**Patriotism of Medical Schools** ... ... ... 428

University of Birmingham.

**The Matrons' and Sisters' Department** ... 429

Head-Mistresses and the Nurse.
Round the Hospitals.
Food Control and the Matrons—The Matrons and Miss Rundle—Mr. Peter Reid—Plain Speech Essential to Success—The Stir of the College—The Rattle Nurse.

**Matrons' and Sisters' Appointments** ... ... 431

**Some War Problems in Food** ... ... ... 431

The Bread Ration.

**The Editor's Letter-Box** ... ... ... ... 432

Nursing Problems in Ireland.

**What One Secretary is Thinking** ... ... iv

"It's a Long, Long Trail."

**Gifts and Bequests** ... ... ... ... ... iv

**Parliament and the Profession** ... ... ... iv

**The Institutional Worker** ... ... (Suppl.) 1

